# Prognostic value of impulsivity in treatment outcomes in patients with alcohol use and/or cocaine use disorder in a rehabilitation programme

**DOI:** 10.4102/sajpsychiatry.v24i0.1297

**Published:** 2018-10-24

**Authors:** Susanne Y. Young, Erine Brocker, Mandi Broodryk, Soraya Seedat

**Affiliations:** 1 Department of Psychiatry, Faculty of Medicine and Health Sciences, Stellenbosch University, South Africa

## Abstract

**Introduction:**

Impulsivity is linked to factors that are negatively correlated with drug and alcohol use. Individuals with substance use disorder (SUD) often suffer from cognitive deficits and, additionally, have high levels of impulsivity. Studies show that cognitive deficits are associated with lower self-efficacy (SE), and the latter is considered an important indicator of SUD management and treatment outcomes. The relationship between impulsivity and SE, however, remains unclear. This prospective study examined impulsivity as a prognostic indicator for SE in SUD populations admitted for inpatient treatment.

**Methods:**

Fifty individuals, aged 18–61, with either a cocaine use and/or alcohol use disorder diagnosis were examined within 72 hours of (1) the start and (2) completion of treatment.

**Results:**

Impulsivity was a significant predictor of SE. Duration of abstinence (in days), estimated intelligence, global assessment of functioning (GAF) and patient age explained 16% of the variance in the change in SE at discharge. After including impulsivity in the regression model, the total variance explained by the model was 28% (*F* [5.505] = 3.47, *p* = 0.01). Impulsivity explained an additional 12% of the variance after controlling for the above variables (*R*^*2*^ change = 0.12, *F* change [4.45] = 7.206, *p* = 0.01).

**Conclusion:**

Impulsivity is a significant predictor of SE following an 8-week impatient treatment programme for individuals diagnosed with SUD. To our knowledge, this is the first study to demonstrate that impulsivity holds prognostic value in respect of the change in SE after inpatient treatment of individuals with SUD. Based on our findings, replication studies are warranted.

